# Animal Chemistry, with Reference to the Physiology and Pathology of Man

**Published:** 1846-10-01

**Authors:** 


I. Animal Chemistry, with reference to the Physiology and Pathology of Man. By Dr. *Franz Simon.* Translated and edited by *George E. Day,* M.A. & L.M., Cantab., Licentiate of the Royal College of Physicians.

II. Dr. Day's Reports on the Progress of Animal Chemistry, in *Ranking's* Half-yearly Abstract of the Medical Sciences. London, 1845-6.

III. Grundlinien der Physiologischen und Pathologischen Chemie. Fur aerzte und Studirende. Von Dr. *Hermann Hoffmann,* mit einer Tafel Abbildungen. Pp. 340. Heidelberg, 1845.

Elements of Physiological and Pathological Chemistry, designed for Practitioners and Students. With a Plate.

IV. A Practical Manual, containing a Description of the General, Chemical, and Microscopic Characters of the Blood and Secretions of the Human Body, as well as of their Components, including both their Healthy and Diseased States, with the best Method of separating and estimating their Ingredients. Also, a succinct Account of the various Concretions found in the Body and forming Calculi. By *John William Griffith,* M.D. F.L.S., Licentiate of the Royal College of Physicians, London, Honorary Member of the Philosophical Society of St. Andrews, and Senior Physician to the Finsbury Dispensary. Small 8vo, pp. 168. London, 1846.

For the work that stands foremost in the above list we are indebted to the Sydenham Society, and we believe that we are merely echoing the feelings of the great majority of its members in stating that it is the most original, and at the same time one of the most valuable books that has yet appeared under their auspices. Six years ago, all that was known of animal chemistry was to be found in the last volume of the great work of Berzelius, occupying something less than 300 pages. Here we have two goodly volumes, embracing no less than 950 pages, and literally crammed with analyses made for the most part by German and French chemists since that period. As it forms a much more complete system of animal chemistry than the works either of Hoffmann or Griffith, we shall adopt

Simon's order and arrangement, and merely notice the other volumes in the elucidation of some few special points. In the first place, we must premise that the Translator and Editor, Dr. Day, has added such a large amount of valuable matter as almost to entitle him to the claim of co-authorship with Simon; the additions to the first volume amounting to about 150, and in the second, considerably exceeding 200 pages. We feel bound in common justice to make this observation, because we believe that very few of our readers can form an adequate idea of the extreme labour incurred in wading through the extensive regions of foreign periodical literature. With this preliminary remark we proceed without further comment to Dr. Day's Introduction, which occupies the first 86 pages of Vol. I., and is devoted to the chemistry of the proximate constituents of the animal body. These are, as usual, divided into the mineral and the organic. The mineral are arranged in three groups, comprising—1. Those which are of service in the animal body, in consequence of their physical properties—water, phosphate of lime, carbonate of lime, phosphate of magnesia, and fluoride of calcium. 2. Those which are useful by their chemical properties—hydrochloric acid, chloride of sodium, carbonate of soda, phosphate of soda, chloride of calcium, chloride of iron, and iron occurring in some unknown condition, as in hæmatin. And, 3. Those which being only incidentally present, may be eliminated without exerting any unfavorable effect on the economy; as, for instance, chloride of potassium, alkaline sulphates, carbonate of magnesia, manganese, silica, alumina, arsenic, copper, lead, and the ammoniacal salts.

The organic constituents are arranged in two groups, the former embracing the nitrogenous, and the latter the non-nitrogenous matters; in the first are considered protein and its compounds—albumen, fibrin, and casein; pepsin, ptyalin, gelatin, pyin, extractive matters, colouring matters, bilin, urea, uric acid, hippuric acid, uric oxide, and cystin; in the second, there are arranged the animal sugars, fats, and non-nitrogenous organic acids, namely, lactic, oxalic, and acetic acid. Of protein and its compounds we find a more comprehensive notice than in Hoffmann's Elements, or Griffith's Manual. Why Dr. Griffith has assigned to protein the formula $C_{40}\ H_{30}\ N_5\ O_{12}$, instead of allowing it an additional equivalent of hydrogen, we know not. We were at first inclined to believe it a misprint for the ordinary formula $C_{40}\ H_{31}\ N_5\ O_{12}$, but finding it originally in page 3, and repeated in the two following pages, we conclude that it is based on some assumed reason. Protein forms, as probably most of our readers are aware, two distinct oxides, viz. the *binoxide*, which is left in an insoluble form, after fibrin has been boiled with water for some hours; and the *tritoxide*, which is formed by boiling fibrin or albumen in water for many hours, precipitating by acetate of lead, and neutralizing the solution by ammonia. After collecting the lead by sulphuretted hydrogen, the tritoxide may be obtained by evaporating the solution. These oxides are continually being produced in the blood, and occur in great excess in inflammation. In fact, the process of inflammation is regarded by Mulder, and we believe correctly, as a higher grade of oxidation. "The albumen of the blood, which furnishes only tritoxide by ebullition, probably takes no part in this change; we may conclude that it is effected by the fibrin alone, which, as we know, absorbs oxygen from the air, and is with so much comparative facility converted into binoxide and tritoxide of protein. During the height of inflammation there is, as we have already mentioned, a great excess of these oxides in the blood. Respiration may consequently be regarded as a true oxidation of the blood, or rather of the protein; and in inflammation, in which the blood contains a greater quantity of binoxide and tritoxide of protein than in the healthy state, this body becomes more thoroughly oxidized. Hence it is, that in acceleration of the act of respiration—in fevers, for example—inflammation so easily supervenes after any violent or sustained efforts. Every paroxysm of fever must necessarily cause the formation of a greater quantity of oxidized protein in the system, and every augmentation in the amount of oxidized protein must produce inflammation, which in its turn may determine fever. Hence it also happens that stimulating foods and drinks which quicken the respiration, or cold air which introduces more oxygen into the lungs, often give the first impulse to the development of inflammation in the organism. The buffy coat is formed when the oxides of protein predominate in the blood; when they accumulate in any particular part of the system, local inflammation is the result. In the latter case, morbid products, as for instance false membranes, are evolved, which are found on analysis to be composed of oxidized protein. Now, inflammation must be combatted by endeavouring to diminish the quantity of tritoxide of protein, and to hinder its formation in the lungs. Venesection proves antiphlogistic by directly diminishing the tritoxide of protein: increased secretion of the alimentary canal indirectly produces the same effect by accelerating the change of substance in the body, and consequently also the consumption of a greater quantity of protein and its oxides." Vol. 1, pp. 12, 13, *Note.*

Many of our readers may not be aware that Mulder's protein-theory, which for the last six or eight years has been regarded as fully established, has, during the last few months, been fiercely assailed by the Giessen school—by Liebig himself, and by Laskowski, one of his pupils—who assert that it is impossible by Mulder's directions to isolate a matter altogether devoid of sulphur, and that his protein is altogether hypothetical. For our own part, we place great reliance on the accuracy of Mulder, and shall, at all events, stick by the protein-theory till we see his reply to these attacks.

Albumen, fibrin, casein, pepsin, and ptyalin, are next discussed, and the reader will find a considerable amount of information on these subjects not to be met with in any other English work. Gelatin is considered by Dr. Day under two heads, as chondrin and glutin—an arrangement, we believe, first adopted by Müller. "The extreme facility with which tannin precipitates gelatinous matter gives a clue to the medicinal action of astringent drugs in the human organism. They at once form insoluble compounds, (for tannin acts similarly on the protein-compounds,) and do not enter the blood; and this is the reason of their being comparatively innocuous. According to Mulder, a less amount of tannin than is contained in one ounce of cinchona bark would, if conveyed directly into the blood, cause instantaneous death." In page 28 we find an ingenious hypothesis (for we can call it nothing more) regarding the origin of glutin. As no traces of it have ever been discovered in the vegetable kingdom we cannot suppose that, like protein, it arises from that source. In all probability it is formed by the action of alkalies on protein; since we know that protein submitted to such influences yields products which in their chemical composition approximate closely to glutin, and that the blood is sufficiently alkaline to effect such or similar modifications. The author then proceeds to illustrate by symbols a possible mode by which glutin and water may be formed from protid and erythroprotid by the ammonia which the alkali of the blood evolves from the protein-compounds, with the co-operation, in the lungs, of the atmospheric oxygen. In the present state of organic chemistry, it is impossible in most cases, as the author himself allows, to state with certainty how changes such as these take place. We can only indicate the possible and the most probable methods. That the gelatinous tissues are evolved from protein-compounds, in some manner or other, cannot admit of a doubt. From what other source can they be derived in the chick, but from the protein-compounds of the egg?

Passing over nine pages devoted to the consideration of the extractive matters, we arrive at the colouring matters of the blood, bile, and urine. The following extract affords a fair illustration of the style of this portion of the work. We select it because it clearly disproves the assumed connection between the red colour of the hæmatin and iron.

"Mulder has carefully examined the action of chlorine on hæmatin. He found that if a current of chlorine be transmitted through water containing hæmatin in suspension, the iron leaves the other elements and forms a chloride of iron, while the atom of metal thus removed is replaced by six atoms of chlorous acid, and a compound is formed, which is represented by the formula $C_{44}\ H_{22}\ N_3\ O_6 + 6\ Cl\ O_3$. During this process the red colouring matter is destroyed, and the new compound appears as a white flocculent precipitate. It must not, however, be assumed from this experiment, that the red colour of the blood is dependent on the iron, for that constituent may be removed from the hæmatin, without materially affecting its tint, as may be shown in the following manner. Let some dried blood be mixed with concentrated sulphuric acid, and after standing for some days let water be added. Hydrogen gas is evolved by the action of the acid on the dried blood, and sulphate of the protoxide of iron is formed. If the blood after this process, be carefully washed, a mixture of alcohol and sulphuric acid will extract from it red hæmatin in combination with sulphoproteic acid, but perfectly free from iron. Van Goudoever has reduced the following formula for this compound:

$$\begin{aligned} C_{84}\ H_{57}\ N_8\ O_{22},\ SO_3 = {} & C_{44}\ H_{22}\ N_3\ O_6 \text{ (the organic part of hæmatin),} \\ & + C_{40}\ H_{31}\ N_5\ O_{12},\ SO_3 \text{ (sulphoproteic acid),} \\ & + 4\ HO. \end{aligned}$$

Although this experiment affords conclusive evidence that the red colour of the hæmatin is not dependent on the iron, yet this metal is very firmly combined with the four organic elements of this constituent. Well prepared hæmatin may be submitted for several days to the action of dilute hydrochloric or sulphuric acid without the iron diminishing in the slightest degree.

"Hæmatin treated in this manner, left after incineration 9·40$\frac{0}{0}$* of peroxide of iron, the amount that is always yielded by well purified hæmatin. The condition in which the iron exists in hæmatin (whether as an oxide, a carbonate, a carburet, or in the metallic state) has long been disputed. The probability of its existence in a metallic state is strongly supported by the evolution of hydrogen that occurs

* This notation indicates per-centage.

when the clot is digested in sulphuric acid, and water is added; and Mulder suggests that this metal probably exists as an integral constituent of hæmatin, in just the same manner as iodine occurs in sponge, sulphur in cystin, or arsenic in the kakodyl series.

"Numerous attempts have been made with the view to ascertain the proportions in which hæmatin and globulin combine, but the results have been very discordant. According to Berzelius, the hæmatoglobulin of human blood contains 100 parts of globulin, and 5·8 of hæmatin. Simon found the ratio to be 100 of globulin to 6·5 of hæmatin in the blood of a healthy young man, and 100 of globulin to 5·3 of hæmatin in the healthy blood of a stout girl. In disease, the variations are much greater. Simon has found as the limits 8·5 and 3·3 of hæmatin, corresponding to 100 of globulin. Regarding the origin of hæmatin, it must clearly be generated in the organism, since it does not exist in the vegetable kingdom. Mulder conceives that it is generated from the normal constituents of the blood in the course of the circulation. Its destination also is obscure. In common with all the constituents of the body, it must be generated, consumed, and reproduced; but in respect to the actual metamorphoses that it undergoes in the organism, or their object, we are perfectly in the dark. Mulder suggests that the products of the decomposition of hæmatin may be possibly traced to the bilifulvin of the bile. Hæmatin may be known, both in its coagulated and soluble state, by its colour. When combined with globulin in the blood-corpuscles, it may be recognized by the microscope. In its coagulated state it may be recognized by its insolubility in water, alcohol, and ether." Vol. 1, pp. 40-42.

Since the publication of the first volume of Simon, the colouring matters of the bile and urine have received considerable attention, the former from Scherer and the latter from Heller and Martin.* A notice of Scherer's researches may be found in the Report on the Progress of Chemistry in Vol. 1 of Ranking's Half-yearly Abstract, p. 343. And an account of Heller's investigations is given in Appendix II. to the 2nd volume of Simon. Heller believes that, in addition to the ordinary colouring matter, there exists a yellow pigment (uroxanthin), which occurs in solution in a very small quantity in healthy urine, but is much increased in certain forms of disease. He states that by oxidation it becomes converted into two other pigments, one of which is of a ruby-red tint (urrhodin), whilst the other is of the colour of ultramarine (uroglancin). These are both insoluble in the urine, forming purple sediments.

"That uroxanthin and its products are derived from urea seems probable, from the circumstance that uroglancin and urrhodin occur in diseases different in most of their characters, but similar in one—the presence of an excess of urea in the blood: thus they are found in Bright's disease, in cholera, and in suppression of urine. Further, when these products occur in considerable quantity, (especially when the blue sediment is spontaneously formed,) there is always much carbonate of ammonia, and very little urea (perhaps mere traces) in the urine, as is often the case in Bright's disease. Finally, Heller has observed the blue tint developed by nitrate of urea artificially prepared and kept moist, and has likewise produced it by adding nitric acid to an old solution of urea partially converted into carbonate of ammonia. The existence of a large quantity of uroxanthin in urine is indicated:

"1. By the clear light-yellow colour of the urine when that secretion is acid, as in cholera, and sometimes in Bright's disease.

---

* Ueber das Urokyanin. Munchen, 1845.

" 2. By the presence of the products of its oxidation, uroglancin and urrhodin, which either of themselves form a violet-coloured sediment, or communicate that tint to a sediment already formed.

" On allowing urine abounding in uroxanthin to stand for some time, it is observed that, after the formation of the sediment has ceased, the fluid from the surface downwards assumes a violent tint, and this change of colour takes place with a rapidity proportional to the amount of carbonate of ammonia produced by the decomposition of urea.

" Hence, on keeping such urine in a high cylindrical glass, three distinct strata are observed; lowermost, a violet sediment; in the middle, yellow and nearly clear urine; and superiorly, a violet or purple turbid layer. On shaking the glass, the whole urine assumes a bluish-green tint, because the urrhodin, formed principally at the surface, becomes converted, by agitation with a full supply of atmospheric air, into uroglancin, which, mixing with the central yellow layer of urine, develops a green tint. The uroglancin thus formed, ultimately settles as a blue powder on the sides and at the bottom of the vessel. Hence there is obviously no fixed proportion between the quantities of uroglancin and urrhodin.

" 3. If much uroxanthin is present, the crystals of uric acid (separated either spontaneously or by the addition of an acid) have a beautiful blue or amythist tint.

" 4. Lastly, if much uroxanthin be present, it may be recognised by the addition of concentrated nitric acid, (ten drops to half an ounce of urine), which at once communicates a brilliant violet colour to the fluid : if a smaller amount be present, the change of colour is developed more slowly. The nitric acid oxydises the uroxanthin, and converts it into uroglancin and urrhodin. Sulphuric and hydrochloric acids act similarly, but with less activity. If albumen is present in urine treated in this manner, it is either precipitated blue at once, or assumes that tint gradually, according to the amount of uroxanthin. This is constantly noticed in Bright's disease on treating urine abounding in uroxanthin, with an acid, and allowing it to stand for a couple of days; uroglancin separates in dark blue crystalline groups, visible to the naked eye, partly on the surface, and partly at the bottom of the vessel.

" To separate the two products of oxidation of uroxanthin, we collect on a filter the sediment thrown down by nitric acid, and agitate it with cold spirit of ·830, which takes up urrhodin (as also does ether); the residue is boiled for some time with spirit of the same strength, until the fluid becomes somewhat concentrated; we thus get a bright blue solution of uroglancin. To exhibit these substances in normal urine, the fluid must be so far evaporated as just to remain liquid. On adding concentrated nitric acid to the cold residue, a crystalline magma of nitrate of urea is at once formed; on adding to this a few more drops of nitric acid (and sometimes even this is unnecessary), it assumes a violet tint. If the crystalline mass is allowed to stand for some time, and is then dissolved in the smallest possible quantity of distilled water, after being left at rest for some time, it deposits a sediment in which urrhodin and uroglancin may be detected either by the microscope, or by extraction with cold, and then boiling spirit.

The action of nitrate of silver on uroxanthin is very singular. On precipitating the chlorine by an excess of nitrate of silver, from urine acidulated with nitric acid, and then carefully neutralizing the filtered liquid by ammonia, there is not only a pale yellow precipitate of phosphate of silver, but the fluid assumes a brown tint, and in a short time there is likewise a brown sediment.

" Heller has not yet succeeded in isolating uroxanthin. Uroglancin associated with urrhodin, occurs in urinary sediments in Bright's disease, and in cases in which urine, abundant in uroxanthin, has become alkaline in the bladder. Heller has noticed it in these sediments forming groups of delicate prisms. It likewise assumes this form when urine, abounding in uroxanthin, is treated with nitric, sulphuric, or hydrochloric acid. In this case it is principally found on the surface of the fluid. When allowed to crystallize from its cold spirituous solution, it forms groups which appear nearly black, but are blue and transparent at the edges. Urrhodin appears to be a less oxidized product of uroxanthin than uroglancin, and usually occurs in much larger quantity. It is most commonly observed in cases in which the urine is alkaline before emission, in consequence of containing much vesical mucus, and its development in such cases is hastened by the addition of nitric acid. The method of isolating it has been already described; Heller has never succeeded in obtaining it from its spirituous solution in a crystalline form. It occurs in granules, which, under the microscope, appear of a beautiful rose-colour. It is resinous in its nature, and burns with a clear flame." Vol. 2, pp. 522-5.

The remarks on bilin in Dr. Day's introduction require considerable modification, to bring them up to the present state of our knowledge on the subject; the reader, however, who takes an interest in the subject, will find in the Half-yearly Reports a full account of Redtenbacher's Analysis of taurin, where it is proved that this substance contains a large amount of sulphur—a fact which entirely overthrows about one-half, if not more of Liebig's views, as laid down in "The Metamorphoses of Tissues," in the second part of his "Animal Chemistry." Thus, in page 155 of the second edition of the English translation, we meet with the following observation. "As a kind of general view of the metamorphoses of the nitrogenised animal secretions, attention may here be very properly directed to the fact, that the nitrogenised products of the transformation of the bile are identical in ultimate composition with the constituents of the urine, if to the latter be added a certain proportion of the elements of water:

$$

\left.\begin{array}{ll}

\text{1 at. uric acid} & C_{10}\,N_4\,H_4\,O_6 \\

\text{1 at. urea} & C_2\,N_2\,H_4\,O_2 \\

\text{22 at. water} & H_{22}\,O_{22}

\end{array}\right\} = \left\{\begin{array}{ll}

\text{3 at. taurin} & C_{12}\,N_3\,H_{21}\,O_{30} \\

\text{3 at. ammonia} & N_3\,H_9

\end{array}\right.

$$

$$

\begin{array}{ccc}

C_{12}\,N_6\,H_{30}\,O_{30} & \qquad & C_{12}\,N_6\,H_{30}\,O_{30}

\end{array}

$$

$$

\left.\begin{array}{ll}

\text{1 at. allantoin} & C_4\,N_2\,H_3\,O_3 \\

\text{7 at. water} & H_7\,O_7

\end{array}\right\} = \left\{\begin{array}{ll}

\text{1 at. taurin} & C_4\,N\,H_7\,O_{10} \\

\text{1 at. ammonia} & N\,H_3

\end{array}\right.

$$

$$
\begin{array}{ccc}
C_4\,N_2\,H_{10}\,O_{10} & \qquad & C_4\,N_2\,H_{10}\,O_{10}
\end{array}
$$

In reference to the metamorphoses of uric acid and of the products of the transformation of the bile, it is not less significant, and worthy of remark, that the addition of oxygen and the elements of water to the elements of uric acid, may yield either taurin and urea, or taurin, carbonic acid, and ammonia.

$$

\left.\begin{array}{ll}

\text{1 at. uric acid} & C_{10}\,N_4\,H_4\,O_6 \\

\text{14 at. water} & H_{14}\,O_{14} \\

\text{2 at. oxygen} & O_2

\end{array}\right\} = \left\{\begin{array}{ll}

\text{2 at. taurin} & C_8\,N_2\,H_{14}\,O_{20} \\

\text{1 at. urea} & C_2\,N_2\,H\,O_2

\end{array}\right.

$$

$$

\left.\begin{array}{ll}

 & C_{10}\,N_4\,H_{18}\,O_{22} \\

\text{Add 2 at. water} & H_2\,O_2

\end{array}\right\} = \left\{\begin{array}{ll}

 & C_{10}\,N_4\,H_{18}\,O_{22} \\

\text{2 at. taurin} & C_8\,N_2\,H_{14}\,O_{20} \\

\text{2 at. carbon. acid} & C_2\,O_4 \\

\text{2 at. ammonia} & N_2\,H_6

\end{array}\right.

$$

$$

\begin{array}{ccc}

C_{10}\,N_4\,H_{20}\,O_{24} & \qquad & C_{10}\,N_4\,H_{20}\,O_{24}\text{"}

\end{array}

$$

All these calculations being thus shown to be based on fallacious data, it is needless to add that this—the most attractive and captivating part of the whole work—the portion almost invariably quoted as showing the true application of Chemistry to Physiology—at once falls to the ground, and our readers may attach what value they think fit to Liebig's "analytical proof that the nitrogenised products of the transformation of bile, namely taurin and ammonia, may be formed from all the constituents of the urine, with the exception of urea—that is from hippuric acid, uric acid and allantoin;" and they may form their own conclusion as to whether "every doubt regarding the possibility of these changes is removed."

This same error respecting the true composition of taurin considerably influences the great tea and coffee principle, laid down in pp. 179-182, of the same volume, and quoted in all tea-total addresses, and in the numerous lectures and treatises on the vegetables affording food to man.* In addition to Redtenbacher's discovery, we may notice Pettenkofer's test for bilin, and Platner's researches, as important additions to this subject, which have appeared since the publication of the first volume of Simon: the former may, however, be found at page 190, and the latter at page 20 of the second volume, or more fully in Ranking, Vol. 1, p. 341, and Vol. 3, p. 313.

The remainder of the Introduction calls for no special remark; it is brought down completely to the date of its publication, and contains a sufficiently copious description of the constituents of the body to facilitate the perusal of the remainder of the work, without wearying the reader with comparatively useless details. Especial stress is in every case laid on the means of diagnosing the substance under consideration—a point of the greatest importance in the examination of the animal fluids.

In addition to the Introduction, the first volume contains two chapters, the first on *the Proximate Analysis of compound Animal Substances,* the 2nd on *the Circulating Fluids.* In Chap. I, we meet with the following observation which we extract, because it affords an useful hint to many young chemists:—"A single isolated analysis is of very little intrinsic value in substances of so varying a nature as the blood and urine. The only method by which we can hope to throw any light upon the leading alterations that occur in these fluids is by the results obtained from a series of analyses; and if we were desirous of merely ascertaining so simple a fact as the determination of the pathological states in which either an excess or deficiency of fibrin and blood-corpuscles occurs in the blood, and the relation that exists between such pathological states and such modifications of the vital fluid, science would be more benefitted by the investigation than by the performance of a few very perfect analyses, which did not tend to elucidate any particular point." Simon then proceeds to give a preliminary sketch of the course that should be adopted if a fluid of whose nature we are ignorant, be placed in our hands for analysis.

"Such a fluid may contain,

---

* See "Fruits and Farinacea, the proper Food for Man," by J. Smith, 1845—or Dr. Lankester's excellent little work on "The Vegetables yielding Food to Man," forming one of Knight's weekly volumes.

I. The *protein combinations,* fibrin, albumen, casein, globulin.
II. *Pyin.*
III. *Extractive matters:* water-extract, spirit-extract, alcohol-extract, and their proximate constituents.
IV. *Sugars:* Diabetic sugar, and sugar of milk.
V. *Bilin,* with the products of its metamorphoses.
VI. *Urea.*
VII. The *Fats:* olein, stearin, margarin, butyrin, cholesterin, and serolin.
VIII. *Colouring matters:* the pigments of the blood and bile.
IX. The *acids* of the animal body:
  α. Fatty acids.
  β. Other organic acids.
  γ. Inorganic acids.
X. The *bases* of the Animal body." Vol. 1, p. 90.

The ten succeeding pages are devoted to the consideration of this subject, and contain an admirable code of laws for the qualitative determination of all the substances mentioned in the above scheme.

The 2nd Chapter is the longest in the whole work, extending over 259 pages, and embracing the consideration of the blood, lymph, and chyle.

Following Simon's arrangement, we shall consider—

1. *The general physical relations of normal blood.*
2. *Its general chemical relations.*
3. *Its chemical physiology.*
4. *Its special chemistry.*
5. *Its relations to physiology.*

And 6. *Diseased blood.*

In the section on the general physical relations of the blood, we meet with all that is known regarding its colour, specific gravity, and temperature. Considerable additions, especially from the papers of Gulliver and Owen, might have been made to the microscopic analysis of the blood, and we are inclined to believe from the manner in which almost every other part of the work has been completed by the editor, that he was deterred from interfering with this department, in consequence of the Sydenham Society having entrusted Mr. Gulliver with the editing of Hewson's works —a volume in which microscopic observations on the blood would find a more natural place than in a work on physiological chemistry: or it may be that he differed in opinion from his author in this point, and did not see what bearing the size of the blood-corpuscles of apes, cats, mice, ducks, geese, and pigeons, had upon "The Chemistry of Man." For our own part we think Dr. Day has done wisely; we only mention the omission because some, into whose hands the work may fall, might deem that all our knowledge of this subject was embraced in pp. 103-4 of Simon. The following remarks on the peculiar forms occasionally presented by blood-corpuscles will be read with interest:—

"The blood-corpuscles do not always present a regular nummular and flattened appearance; they are sometimes plicated and bent in. The cause of this phenomenon is not known, but it is probably due to a contraction of the capsule at different points. One of the most peculiar of these forms is that in which the edge of the blood-corpuscle appears as if it were studded with minute pearls. In the blood of a patient suffering from Bright's disease, I found that nearly all the blood-corpuscles had undergone this modification. On the addition of a solution of muriate of ammonia, the appearance it presented under the microscope was very striking. I immediately made a counter-experiment with my own blood, but it did not exhibit the phenomenon in question. Ascherson has offered the following explanation of this peculiarity in the form of the corpuscles, viz. that it is due to the exudation of fat which exists in a fluid state in the blood-corpuscle. In opposition to this view, it may be urged, that if each individual corpuscle contained a separable portion of fat (however minute it might be), we should obtain in our analyses a much larger quantity of fat than in reality we do. It is true that the dried clot yields a larger proportion of fat than an equal weight of serum, but the difference is by no means so striking as it would have been if Ascherson's theory were correct. Hünefeld has observed a similar appearance on treating the blood-corpuscles of the frog with putrid serum, in which granules were present. The granules seemed to form a sort of girdle round the corpuscle, and he conceives that they penetrated into minute depressions upon the surface of the capsule. If this statement be correct, it is strongly opposed to the observations of Ascherson and Wagner respecting the lubricity and evenness of the blood-corpuscles. On mixing the blood of a carp with a solution of sugar, and on the cautious addition of water, I observed that the blood-corpuscles assumed a stellar appearance. On treating frog's blood with bilin, an agent which usually dissolves the corpuscles, I observed that some of them resisted this action for a considerable period, and ultimately assumed a pyriform appearance, while others became narrowed at the centre, and extended at both extremities. Others, again, seemed to undergo an internal change, and appeared as if their inner surface were studded with minute vesicles. Hünefeld made a similar observation on treating frog's blood with carbonate of ammonia. The same chemist observed a remarkable peculiarity in the corpuscles of human blood, on the addition of sulphate of quinine. In the course of a few minutes they assumed an irregular, angular form, and appeared as if their sides were drawn together. Schultz has made the following important microscopic observation. On examining the blood-corpuscles of a salamander which had been suffocated in carbonic acid gas, they were found to be of a darker colour than usual; the darkness was particularly marked on some spots, so that they exhibited a sort of chequered appearance. On shaking the blood with oxygen gas, the corpuscles became brighter and more transparent." Vol. 1, p. 105-6.

We now arrive at the second section—*on the general chemical relations of the blood.* The chemical relations of the corpuscles are first considered. The principal labourers in this department are Müller, Schultz, Hünefeld, and Simon, who have observed under the microscope the apparent effects produced on them by the addition of numerous chemical and medicinal agents. There can be no doubt that enquiries of this nature, properly conducted, must ultimately lead to important practical results. With regard to the colouring matter, Hünefeld believes that it exists in an insoluble form, attached to the inner surface of the capsule. Simon opposes this view; he observed that it "is contained in all probability in a state of solution in the corpuscles, an opinion which is also supported by Müller, Schultz, and Reichert. If, on the examination of frog's blood, one corpuscle be observed to move over another, the lower can be distinctly perceived through the upper one. Moreover, the instantaneous solution of the corpuscles by means of bilin, supports this view, for, if their contents were gelatinous or solid, the act of solution would be observed to progress from the circumference to the centre, and would admit of being observed by the microscope." The general chemical relations of the nuclei, and of the *liquor sanguinis,* call for no special remark, neither do the observations on the retardation and acceleration of the coagulum.

The *chemical physiology of the blood* forms a highly important section, and we shall devote some space to its consideration.

*The formation of the blood,* and the *forces that circulate it,* belonging strictly to physiology, we proceed to *the process of respiration.* Dr. Day has made extensive additions to this department, incorporating the recent investigations of Andral and Gavarret on the quantity of carbonic acid exhaled *per horam,* those of Scharling on the same subject, and those of Brunner and Valentin on the composition of expired air. From the experiments described in the text it follows "that the quantity of carbonic acid expired is very variable, and that it may be altered by many circumstances. Hunger and rest diminish, satiety and labour increase it. It is greater during the day than during the night, in the proportion of 1·237 to one. If the expired carbonic acid be estimated in relation to the weight of the body, it is found that children give off a proportionally greater amount of this gas than adults. In some forms of disease, the amount of expired carbonic acid falls below the standard; it seems, in a state of health, to vary directly with the activity of the circulation." In connexion with this subject the reader would do well to consult Hannover's Memoir on the Amount of Carbonic Acid exhaled in Disease, or the notice of it in Day's Report on Chemistry, in the last volume of Ranking's Abstract. A considerable space is devoted to *animal heat, and the changes undergone by the blood;* and although we are not prepared to express our conviction of the entire accuracy of the more minute details, we cannot refrain from recommending this section to the attentive perusal of our readers. We regard it as the most successful attempt yet made to apply chemistry to the elucidation of animal physiology. Simon evidently attached much importance to this portion of his work, as he especially alludes to it in his preface, where we find his views on this subject summed up in the following terms:—

"The blood is subjected to a continuous metamorphosis, which may be regarded as the expression of its vitality. The nutrition of the peripheral system is effected by the liquor sanguinis, not by the blood-corpuscles. The liquor sanguinis affords nutriment to the cells and organs, which possess an inherent power of selecting proper material, or of forming it from non-homologous matter, at the same time secreting the products of decomposition. The principal nutritive matters in the liquor sanguinis are albumen, fibrin, and fat. The chief products of this metamorphosis are the extractive matters and lactic acid, which occur in the excretions, especially in the urine. Urea, bilin, and carbonic acid are either not products of the metamorphosis of the blood during the act of nutrition in the peripheral system, or at most they are only in part formed by it. They must be regarded as products of the vital energy of the blood-corpuscles, which doubtless possess the same power of attracting nutriment, and of throwing off decomposed products as other living cells. The proper nutriment of these corpuscles is oxygen, albumen, and probably also fat, which are furnished them by the liquor sanguinis. The most important products of their metamorphosis are carbonic acid, urea, fibrin, extractive matters, and very probably some of the constituents of the bile. The leading and most important object of this vital energy of the blood-corpuscles is the production of animal heat, without which every function of the organism, nay, even life itself, would be instantaneously annihilated. The production of animal heat is due to the combination of oxygen with the carbon of the globulin; the principal products of this reaction are carbonic acid and urea, or uric acid, (which is excreted as a substitute for urea in most of those classes of animals in which elliptic blood-corpuscles occur.) The urea excreted may thus be regarded as a measure or equivalent of the animal heat developed. The production of blood-corpuscles and the formation of blood are intimately connected with nutrition; when the food is too scanty and insufficient, the amount of blood, and especially of blood-corpuscles, is diminished; when the nutriment is proper and abundant, the reverse takes place. In the former case, therefore, the vital energy is depressed, the secretions and excretions are diminished, and the animal heat sinks; while in the latter case, exactly the reverse is observed. In the normal state there is an equilibrium preserved between the production and consumption of blood-corpuscles. The food is prepared, and to a certain extent assimilated, before it enters the blood. The vital energy of the blood-corpuscles continues even during a perfect abstinence from food, and carbonic acid and urea continue to be formed, although their amount gradually diminishes in a direct ratio with the diminution of the blood-corpuscles. Moreover, the amount of carbonic acid and the formation of urea are lessened by a torpid, and increased by an excited circulation; and in proportion to the amount of corpuscles and to the rapidity of the circulation, so much the higher is the animal temperature. Thus in birds we observe a high temperature, and the reverse in the amphibia. In chlorotic, and also in very aged persons, we find a low temperature, and a diminished excretion of urea; while in inflammatory diseases, and after prolonged corporeal exertion, the temperature rises, and there is either a relative or an absolute increase of urea, in the former case, even in the absence of all nitrogenous food. The capillary and cutaneous systems tend to regulate an excessive rapidity of the circulation, and to prevent the animal heat from exceeding a certain limit. If we only knew whether, and in what manner, the pulmonary exhalation is changed in various diseases, (especially in relation to the amount of carbonic acid contained in it,)—whether the carbonic acid always increases relatively with the urea or in certain cases with the uric acid,—and if further we possessed experiments illustrative of the effects of diseases, and of varied diet on the bile, we should then have a more solid basis than we now occupy, on which to found our chemical inquiries, while the acquisition to the science of medicine would be positive and incalculable. The questions here involved must however, unfortunately, at the present time, be regarded as unanswerable. We cannot doubt that the pulmonary exhalation does vary, under different circumstances, in the amount of carbonic acid; for instance, more carbonic acid is exhaled during prolonged corporeal exertion than when the body is in a state of repose, although, as far as I am aware, no experiments on this subject have yet been instituted. We have, however, conclusive evidence that the amount of urea is increased under these circumstances." Vol. 1, pp. xi—xiv.

We now arrive at *the special chemistry of the blood.* The various methods of analysing this fluid are given at considerable length; we have the methods of Berzelius, Lecanu, Denis, and Simon; and to these Dr. Day has added the method of Figuier, which we regard from its simplicity, as the best in all ordinary cases when we wish to determine only the most important constituents. The method adopted by Andral and Gavarret for analysing coagulated blood, is given further on, in the section on diseased blood: we cannot see why it should be separated from Simon's method given in p. 190. The method of Figuier is based on the fact that, after the addition of a neutral salt to defibrinated blood, the globules do not (as before) pass through filtering paper. On the addition of two parts of a solution of sulphate of soda of spec. grav. 1·130 to one of blood, Figuier found that the whole of the corpuscles remained on the surface of the filter. The following are the steps of his analysis. The fibrin is removed by whipping, dried, and weighed; the weight of the corpuscles is ascertained by the method indicated; and that of the albumen by coagulating, by means of heat, the filtered solution. The proportion of water is known by evaporating a small portion of blood whose weight has been previously determined.

We proceed to "*The healthy blood in relation to physiology.*" The distinctions between arterial and venous blood are thus summed up:—"Arterial blood contains less solid residue generally than venous blood: it contains less fat, less albumen, less hæmatin, less extractive matters and salts than venous blood. The blood-corpuscles of arterial blood contain less colouring matter than those of venous blood." Dr. Day adopts Mulder's theory to account for the difference of colour between arterial and venous blood. The oxides of protein formed in the act of respiration solidify round each corpuscle, making the capsule thicker and better qualified to reflect light. Each corpuscle of arterialized blood is thus invested with a complete envelope of buffy coat, which gradually contracts and speedily forms cupped or bi-concave surfaces favorable to the reflection of light. On reaching the capillaries the coating of the oxides is removed for purposes connected with the nutrition of the organism, and the corpuscles losing their opaque investment and their cupped form, can no longer reflect light, and the blood assumes a venous tint.

The peculiar characters of the blood of the portal, hepatic, and renal veins are then noticed, and we regret that want of space precludes us from quoting the important physiological results deduced from the analysis of these varieties of the circulating fluid.

Simon has made two analyses of healthy venous blood, yielding the following results:—

In 1000 parts there were contained:—

| | A Male aged 17 years. | A Female aged 28 years. |
|---|---|---|
| Water | 791·900 | 798·656 |
| Solid residua | 208·100 | 201·344 |
| Fibrin | 2·011 | 2·208 |
| Fat | 1·978 | 2·713 |
| Albumen | 75·590 | 77·610 |
| Globulin | 105·165 | 100·890 |
| Hæmatin | 7·181 | 5·237 |
| Extractive matters and salts | 14·174 | 9·950 |
| Colouring matter in 100 parts of blood-corpuscles | 6·3 | 5.2 |

From these analyses it appears that healthy blood "contains about 30% of solid constituents; not much more than 0·2% of fibrin, and about an equal quantity of fat; the blood-corpuscles considerably exceed the albumen in quantity, and contain about 5·7% of colouring matter." The analyses of Lecanu and Denis are then quoted, and the Editor has added others by Nasse, Becquerel and Rodier, Marchand and Enderlin. Passing over a few pages devoted to the consideration of the effects of sex, temperament, and age on the blood, we arrive at its *pathological conditions.* The extent to which the different constituents may vary is forcibly shown in the following table, drawn up solely from the original analyses of Simon,

| | | |
|---|---|---|
| The water may vary from | 880·0 to | 750·0 |
| Fibrin | 9·1 | a trace. |
| Fat | 4·3 | 0·7 |
| Albumen | 131·0 | 55·1 |
| Globulin | 106·6 | 30·8 |
| Hæmatin | 8·7 | 1·4 |
| Extractive Matters and Salts | 16·5 | 7·6 |

Moreover these must not be regarded as the extreme limits. Thus Andral and Gavarret found the fibrin amount to 10·5 ; Rindstropf to 12·7 (Simon p. 262), and Popp in a case of articular rheumatism found it as high as 13·3 (Day's Report in Vol. 3. p. 307 of Ranking's Half-yearly Abstract.) Diseased blood is arranged in four divisions:—

1. *Hyperinosis* (υπερ and ις, ινος fibre) in which there is an excess of fibrin and a corresponding diminution of the corpuscles.

2. *Hypinosis*, in which the quantity of fibrin is less than in healthy blood, or if it amounts to the normal quantity, its proportion to the blood-corpuscles is less than in a state of health.

3. *Spanæmia* (from σπανος poor, and αιμα blood), in which the blood contains an excess of water, no constituent exceeding the normal average.

4. *Hetero-chymeusis* (from ἑτερος and χύμευσις chylification) which includes those states of the blood in which a substance is present that does not exist in the normal fluid—when, for instance, the blood contains urea (in appreciable quantity), sugar, biliphæin, fat, or pus. The last division is altogether artificial and merely adopted for convenience, since the only common property possessed by this class is, that the composition of the blood is here qualitatively changed, whilst in the three others it was merely altered quantitatively.

This is undoubtedly the most suggestive portion of the work. It is an enormous collection of isolated facts from which many valuable deductions of a practical character may be drawn. A reference to the description of the blood in chlorosis will sufficiently illustrate our meaning. A supplement embraces the following subjects:—blood during pregnancy, menstrual blood, the lochial discharge, and the blood of animals. It extends over fifteen pages, and is almost entirely from the pen of the English editor. The chapter, and with it the volume, terminates with the consideration of the lymph and chyle. It contains, we believe, all that is known on the subject, with the exception of Gulliver's Microscopic Investigations, but still leaves the subject in a very unsatisfactory and imperfect state.

The Third Chapter—the commencement of the 2nd volume—is devoted to the consideration of the *secretions of the chylopoietic viscera, and the theory of digestion.* There is nothing in the observations on the saliva, or pancreatic fluid requiring comment. The bile is described at considerable length, and Dr. Day has added the following remarks on its uses.

"That the bile is not merely an excrementitious fluid, intended to remove effete matters from the blood, but that it is a secretion essential to the animal economy, was rendered almost certain by the experiments of Berzelius, Theyer and Schlosser; which showed that the human fæces contained much too small a quantity of a substance resembling bile to justify the idea, that it is evacuated in this manner. A further proof that the bile is absorbed, and not excreted, is afforded by an examination made by Enderlin, of the ash yielded by the contents of the different portions of the intestinal canal of a hare. He found that the ash from the contents of the duodenum *alone* effervesced on the addition of an acid, thus showing that the choleate of soda (which yields the carbonate on incineration) is absorbed before reaching the jejunum. Schwann has recently established this opinion beyond a doubt, by a series of well-devised experiments on dogs. He tied the ductus communis choledochus, and at the same time formed a fistulous opening in the gall-bladder, by which the bile escaped externally. His most important conclusions are—1st, That when the bile does not get into the bowel, its absence is generally perceptible in dogs about the third day by a marked diminution in weight; and 2ndly, That, unless the channel for the conveyance of bile to the duodenum is re-established, symptoms of deficient nutrition, wasting, debility, &c. ensue, and death is the ultimate consequence."

Few fluids have recently met with more attention at the hands of chemists than the gastric juice, and very few have given rise to more discordant results. Prout showed that it contained free muriatic acid. Tiedmann and Gmelin found both acetic and muriatic acids, and there is no doubt, adds Simon, that lactic acid likewise occurs in it. M. Blondlot has recently published a treatise on Digestion detailing numerous experiments made on dogs, in whom fistulous openings into the stomach were maintained for upwards of two years. His conclusion, deduced from a large number of experiments, is that the acid secretion of healthy gastric juice is owing to the presence of superphosphate or biphosphate of lime, and that the fluid contains no free acetic, hydrochloric, or lactic acid. M. Blondlot's views were speedily attacked by MM. Bernard and Barreswil who "conclude that the acid reaction of the gastric juice is not owing to biphosphate of lime, but arises from a free acid, which is not hydrochloric, or acetic acid." They have always found lactic acid with a minute proportion of phosphoric acid; the latter being a product of the reactions of the lactic acid on the phosphates present. M. Melsens has also examined the gastric juice, and denies the accuracy of Blondlot's conclusions. The latest experiments on the subject are those of Dr. R. D. Thompson. In order to prevent complication of the phenomena, the animals (pigs) were fed on vegetable food alone. His experiments tend to show that no free hydrochloric acid is present in the stomach of animals living on vegetable food, but that the free acid is the lactic. A little acetic acid was also generally present.—*Day's Report on the Progress of Chemistry,* vol. 2, p. 347-51.

It appears, from an observation in a recent number of Heller's Archiv., that Dr. Mack of Vienna is at present engaged in the analysis of the gastric juice and the chemistry of digestion, a patient with gastric fistula affording him ample opportunity for the investigation. It is most sincerely to be wished that his labours may settle the point beyond all further dispute.

The next chapter treats of the Milk, and we believe we may add contains everything known upon the subject. Simon observes that a metastatic or vicarious secretion of this fluid is by no means rare. Dr. Day quotes two very singular illustrative cases—the most convincing we ever met with, since the fluid contained sugar of milk, butter, and casein. In one, the liquid was contained within the coats of the testicle (Vol. 1, p. 65); in the other, it flowed from an abscess in the thigh of a woman who was suckling (vol. 2, p. 521); he has likewise inserted an analysis of the mammary secretion of a *he-goat,* (vol. 2, p. 65.)

The following is Simon's description of the colostrum—the milk secreted immediately after delivery.

"On examining it with the microscope a very large number of fat-globules are seen (some of which are larger than those that occur in ordinary milk) and these are frequently observed clinging to one another; besides these there are granulated, yellow, roundish corpuscles, larger than the milk-corpuscles, which appear to be composed of very minute fat-vesicles; they seem to be peculiar to the colostrum, and were first observed by Donné, who states that they occur in woman's milk till the twenty-eighth day, when the milk loses all the characters of colostrum; I have never succeeded in detecting them after the eighth or tenth day."

From comparative analyses of the colostrum and healthy milk of the same woman, it appears that the former is much the richer of the two in solid constituents, especially in butter and sugar of milk.

The absolute quantity of the casein is also greater, but the ratio of the casein to the solid constituents is less than in ordinary milk. The salts are also increased. The aperient property of the colostrum is probably dependant on the increased quantity of salts and sugar of milk.

The characters of ordinary human milk, the effect of temperament, the changes dependant on nutrition, the changes corresponding with the age of the infant, the modifications produced by disease, and the effects of medicines on the milk, are then considered; and the chapter terminates with the "milk of animals."

In the Appendix (vol. 2, p. 521) we find a notice of some very important analyses recently instituted by Dumas. He shows that, after dogs have been restricted for some time to a purely animal diet, all traces of sugar disappear from the milk.

These experiments bear very forcibly on the much disputed question of the existence or non-existence of lactic acid in the animal fluids.

Chapters V. and VI. embrace the consideration of the secretions of the mucous membrane and the external skin; namely, mucus, pus, and sweat.

The following analysis of arthritic pus is interesting, as showing the general predominance of uric acid in the body in cases of gouty diathesis.

"I have examined the dried residue of the liquor puris of an arthritic person; it was of a grayish yellow colour, containing no membranous shreds, could be easily pulverised, and exhibited no appearance of crystals when examined under the microscope. On heating it with nitric acid, I obtained, after the evaporation of the acid, and more strikingly on the addition of ammonia, a brilliant purple colour, indicating the presence of uric acid beyond a doubt. On triturating this substance with water I obtained a pulpy mass, which, when examined under the microscope, was found to contain numerous epithelium-cells and pus-corpuscles, but no crystals of uric acid. Alcohol extracted 5·4% of fat, consisting chiefly of margaric and oleic acids with a little cholesterin; boiling water took up 52·6%, of which a little fat and extractive matters, with hydrochlorate of ammonia and lactate of soda, were soluble in anhydrous alcohol; and chloride of sodium, extractive matter, and albuminate of soda, in spirit. The remainder was washed with cold water (which extracted very little) and was then dissolved in a faintly alkaline solution. On the addition of hydrochloric acid to this alkaline solution, crystals of uric acid were deposited, and some albumen thrown down from the albuminate of soda: the acid solution then contained hydrochlorate of ammonia and chloride of sodium. The portion insoluble in water yielded on incineration

5 ℥ of ash, consisting of earthy phosphates, with a little peroxide of iron and carbonate of soda; the dried residue of the liquor puris yielded, however, 10 ℥ of ash, composed of carbonate of soda, a little phosphate of soda, carbonate and phosphate of lime, a little chloride of sodium, and traces of peroxide of iron."—Vol. 2, p. 95.

It is not a great many years ago since *pus-tests* were in great fashion; and, when the occurrence of pus in the expectoration was regarded as diagnostic of phthisis, extreme importance was attached to the difference between it and mucus. These tests were mostly based on the chemical relations of the corpuscles towards various re-agents; thus, Grasmeyer's and Donné's tests were founded on the action of the alkalies and their carbonates on pus. Güterbock's test was based on the circumstance that pus, being fatty, burned with a clearer flame than mucus, and there were other tests by Gruithuisen, Young, and others. None of these tests are to be altogether trusted, and the microscope renders them superfluous. If this instrument reveals their apparent presence, and on the addition of acetic acid the corpuscles disappear, leaving only cup-formed nuclei, we may be sure we are examining pus.

The Seventh Chapter extends over 240 pages, containing by far the most perfect history of the urine in health and disease, that has yet appeared in any language. It would lead us far beyond our proper limits to attempt a condensation of this part of the work. It commences with the physical characters and the qualitative and quantitative analysis of the healthy urine. In addition to the analysis of Berzelius, we find five by Simon, three by Lehmann, and others by Becquerel, Marchand, and Day. The physiological relations of the urine are then considered; these are chiefly based on the researches of Liebig on the influence of the salts contained in the food upon the composition of the urine, on those of Lehmann on the influence of diet on this secretion, and on those of Lecanu on the influence of age and sex. The greater portion of this matter has been introduced by the English editor.

We now arrive at the pathological relations of the urine. The chemical changes may be reduced to one of the following forms:—

1. One or more of the normal constituents existing in larger quantity than in healthy urine.
2. One or more of the normal constituents existing in less quantity than in healthy urine.
3. A normal constituent absent.
4. The presence of substances that do not exist in normal urine.

The substances noticed by Simon under the fourth head, are albumen, blood-corpuscles, fibrin, fat, chyle, casein, biliphæin, bilin and bilifellinic acid, sugar, carbonate of ammonia, oxalate of lime, carbonate of lime, cystin, and pus.

The constitution of the urine in different diseases is now considered, no less than 117 pages being devoted to this subject. We select the following observations regarding the determination of the sugar and urea in diabetic urine.

"On treating diabetic urine evaporated to the thickness of a syrup with warm spirit, the mucus, uric acid or urates, and earthy phosphates are precipitated. On evaporating the filtered spirituous solution to the consistence of a thin syrup, and adding anhydrous alcohol, an insoluble semi-fluid mass separates, which, when repeatedly treated with anhydrous alcohol, becomes finally thick and tough.

On dissolving this saccharine mass in warm spirit, and again precipitating it by anhydrous alcohol, it will still be found to contain a certain amount of urea; in fact, I have detected urea after the operation has thrice been effected, and I find that sugar can only be obtained free from urea by allowing it to crystallize spontaneously from its spirituous solution. In consequence of the difficulty of separating these substances, I proceed in the following manner: the solid residue of the urine is first accurately determined; a weighed portion of urine is then evaporated, mixed with spirit, and the solution filtered. The filtered solution is evaporated to the consistence of a syrup, and, when cold, mixed with a sufficient quantity of concentrated nitric acid to allow of a few drops remaining on the surface of the crystalline mass. It must be submitted to a low temperature, and the crystals placed on blotting paper and compressed till they cease to communicate moisture. The fixed salts must be determined from a separate portion of urine. If we deduct from the known quantity of solid residue the portion insoluble in spirit (from which the uric acid is determined), the urea, and the fixed salts, we obtain, as the difference, sugar and alcohol-extract which appears to decrease in diabetic urine in proportion as the sugar increases."—Vol. 2, pp. 297-8.

Urines containing fat, milk, an excess of hippuric acid (of which Dr. Day has collected three singular cases), urostealith, and semen are then considered, and these are succeeded by observations on "urine of peculiar colours," "urine during pregnancy," and on "the passage of medicinal and other substances into the urine." The chapter terminates with "the urine of animals."

There is nothing in Chapters VIII. and IX. to call for especial remark. The former is devoted to the consideration of the secretion of the lachrymal meibomean and ceruminous glands; the latter, to the secretions and fluids of the generative organs, including the seminal and prostatic fluids, the amniotic and allantoic fluids, and the vernix caseosa.

The Tenth Chapter treats of the intestinal excretions. We have full accounts of the meconium and the fæces of infants and of adults, in health and in disease. The diseases especially noticed as influencing the fæces are diabetes, dysentery, enteritis, abdominal typhus, diarrhœa, cholera, enteropthisis and jaundice. The effects of calomel on the evacuations are then considered; and the chapter terminates with the chemistry of vomited matters.

The component parts of the animal body form the subject of the Eleventh Chapter. The article on bone is re-written by the Editor, the matter being chiefly taken from the recent work of Von Bibra on the subject. Teeth, cartilage, cellular tissue, tendon, ligament, skin and hair, are then considered. From the observations of Van Laer it appears that the hair consists essentially of:—

"(1). A connecting medium consisting of a tissue yielding gelatin, and represented by the formula $C_{13}\ H_{10}\ N_3\ O_5$; and (2), Of bisulphuret of protein, $C_{40}\ H_{31}\ N_5\ O_{12}\ S_2$.

"The large amount of sulphur in hair (averaging 5%) is the cause of its colour being affected by various metallic salts. As there is no constant difference in the results obtained by the analysis of hair of various tints, it is to be presumed that the colour is dependent on peculiar arrangements of the ultimate particles."—Vol. 2, p. 419.

In the crystalline lens, Simon finds, in addition to albumen, a peculiar substance closely resembling casein, to which he applies the term crystallin. When the lens becomes opaque (in cases of cataract), it is found to contain an excess of phosphate of lime.

The muscles next claim our attention. Simon observes that, "on making incisions in the warm flesh of an animal just killed, we obtain by pressure an acid fluid, which rapidly coagulates in consequence of the presence of a little fibrin: if the flesh has been kept for some time the fluid obtained by pressure no longer coagulates, although it exhibits an acid re-action." We quote these lines to draw the attention of our chemical readers to the nature of this acid. Hoffman (op. cit., p. 130, note) observes that, from this acid fluid he obtained a zinc-salt, having the crystalline form of the lactate of zinc. The obscurity which rests over the question regarding the existence or non-existence of lactic acid in the animal body is disgraceful to the science of chemistry. The brain, spinal cord, and glands, occupy the remainder of the chapter.

The two remaining chapters are devoted to solid and fluid morbid products. The former contains full instruction for the qualitative and quantitative analysis of the various concretions occurring in the animal body, together with a good account of their physical and chemical characters; it likewise embraces the chemistry of tubercle, scrofula, and cancer, in so far as it is yet known. The latter contains analyses of the fluids in various cysts, the fluids of pemphigus, hygroma, hydrocephalus, ascites, hydrocele, thoracic effusions, the subcutaneous serum in Bright's disease, and the fluid effusions found in the body after death.

There are two Appendices, the former containing the ultimate composition of the proximate constituents of the human organism, the latter embracing the most important additions to animal chemistry made during the course of publication.

The value of the work is much increased by three accurate and well-engraved illustrative plates, and by a very copious and most useful index extending over eighteen pages. The length to which our review has extended, and the numerous extracts we have made, afford sufficient evidence of our opinion respecting its great merits. It is in the highest degree creditable to the Author, the Editor, and the Sydenham Society. We believe that the publication of this work, and of Hasse's Pathological Anatomy (noticed in our last number), will tend—provided always the re-printing of some of the standard old medical works be not forgotten—to instil into the minds of their medical brethren a confidence in the proceedings of the Council, which some of their acts have gone far to undermine, and we most sincerely trust that this may be the case. We cannot, however, help remarking upon the singularity of the fact, that for both these works we have been indebted to the suggestions of their respective editors, and not to any intimate knowledge of their contents by the twenty-four learned gentlemen forming the aforesaid body.

Of Dr. Day's Reports on the Progress of Chemistry in relation to Physiology and Pathology we need merely add, that they are written in the same spirit of research and impartiality which characterises his extensive additions to Simon. Every medical man is expected to be conversant with the recent advances of physiological and pathological chemistry, and these reports afford the only means in the English language of meeting these expectations. We regard them as one of the most important features in Ranking's Half-yearly Abstract.

Hoffman's Elements are clearly written, but present no claim to originality. His principal fault lies in the undue importance he attaches to subjects of little intrinsic value, or, at all events, hardly bearing, even in a remote degree, on Animal Chemistry; thus, nearly six pages are devoted to the glands, for the most part to their development and uses; while the space occupied by the blood—the most important subject in the whole work—is considerably less than ten pages.

Of the manual of Dr. Griffith, his own title-page (a work we opine of no small labour) gives a full, and we may add, a true and correct account. It is the only portion of the book that would bear much abbreviation; indeed, in many points we think that the system of condensation has been carried a little too far. The work commences with the protein and gelatinous compounds and the extractive, and fatty matters. We have already (see page 482) had occasion to notice his introduction of a *new* formula for protein; we must now take him to task for the opposite fault—for retaining *obsolete* chemical opinions; in page 15 we find that "gelatine occurs in the bone, skin, serous membranes, cellular tissue, tendons, ligaments, and ossified cartilages." Gelatine no more *occurs* in these structures than does tritoxide of protein in albumen. *It is obtained* from them by ebullition.

Organic acids next claim our attention—lactic, acetic, hydrocyanic, formic, benzoic, oxalic, oxaluric, and tartaric acids. This list is rather more extensive than need be; the development of hydrocyanic acid in the animal body is more than doubtful; formic and oxaluric acids have never been discovered in any of the secretions, and benzoic acid, when present in the urine, is a result of decomposition; and regarding tartaric acid, which we are told "has been found in diabetic urine in combination with lime," we would suggest that the diabetes had nothing to do with the question, further than that the patient, being thirsty, probably drank *imperial*—a solution of bitartrate of potash—which would fully account for the phenomenon in question.

The rules for the detection and separation of the *inorganic matters* are concise, and at the same time clear; we would however observe, that Dr. Day has given a more delicate test for the presence of carbonic acid (Simon, vol. ii. p. 120) than Dr. Griffith. It would perhaps have been better not to have alluded to the supposed discovery of titanic acid in the renal capsules. There can be little doubt that there was some fallacy in the experiment, and even if it really were there (and from the frequent association of titanium with iron, the thing is not altogether impossible), it must have been altogether an accidental occurrence, and would have afforded no just grounds for the statement that the renal capsules contain titanic acid more than any other part of the body. Dr. Griffith's manual shows that he is well acquainted with the processes he describes; and, if in several points he has not carried his subject to quite the latest date, his readers may at least be assured that he will never lead them into serious error. Considering the limited space he had at his command, most of the subjects he treats of are handled in a very satisfactory manner. We have therefore pleasure in recommending his work as a useful introduction to the more elaborate writings of Simon and Day.

